# Clinical value of combined *SHOX2* and *RASSF1A* methylation in lung cancer diagnosis across tissue and liquid biopsy samples: a systematic review and meta-analysis

**DOI:** 10.3389/or.2026.1876563

**Published:** 2026-08-28

**Authors:** Ting Wang, Jianguo Zhang, Jiayang Wang, Xiaoqun Dong, Qiushi Lin, Leiming Guo, Bo Zhang, Yue Cao, Yining Zhai, Mathhew Hadfield, Khan Hina, Abbas E. Abbas, Hong Ge, Liang Cheng, Hang Xing, Yifei Liu

**Affiliations:** 1 Department of Radiation Oncology, The Affiliated Cancer Hospital of Zhengzhou University and Henan Cancer Hospital, Zhengzhou, Henan, China; 2 Department of Pathology, Affiliated Hospital of Nantong University, Nantong, Jiangsu, China; 3 Precision Health Program, Department of Radiology, College of Human Medicine, Michigan State University, East Lansing, MI, United States; 4 Division of Hematology and Oncology, Rhode Island Hospital, Warren Alpert Medical School of Brown University, Providence, RI, United States; 5 Division of Cardiothoracic Surgery, Rhode Island Hospital, Warren Alpert Medical School of Brown University, Providence, RI, United States; 6 Department of Pathology and Laboratory Medicine, Warren Alpert Medical School of Brown University, The Legorreta Cancer Center at Brown University, and Brown University Health, Providence, RI, United States; 7 Department of Surgery, Warren Alpert Medical School of Brown University, The Legorreta Cancer Center at Brown University, and Brown University Health, Providence, RI, United States

**Keywords:** biomarker, DNA methylation, early detection, liquid biopsy, lung cancer, molecular diagnosis, RASSF1A, SHOX2

## Abstract

Lung cancer remains the leading cause of cancer-related mortality worldwide, with poor prognosis in advanced stage diseases. Although early diagnosis has the potential to improve patient outcomes, current diagnostic methods remain suboptimal, highlighting the need for accurate molecular biomarkers. We systematically reviewed 49 studies to evaluate the diagnostic performance of SHOX2 methylation, RASSF1A methylation, and their combined panel for lung cancer detection. The combined SHOX2/RASSF1A methylation panel demonstrated a pooled sensitivity of 77.8% (95% CI: 72.3%–82.5%) and specificity of 89.0% (95% CI: 86.6%–91.1%), with an HSROC area under the curve (AUC) of 0.916. SHOX2 methylation alone yielded a sensitivity of 69.4% and specificity of 91.7%, whereas RASSF1A methylation showed lower sensitivity (45.7%) but the highest specificity (93.8%). Pairwise comparisons demonstrated that the combined panel significantly improved sensitivity compared with either SHOX2 or RASSF1A alone while maintaining specificity comparable to SHOX2, although lower than that of RASSF1A. Subgroup analyses showed that assay method and pathological subtype contributed to differences in pooled sensitivity, whereas leave-one-out sensitivity analyses confirmed the robustness of the pooled estimates. In conclusion, the combined SHOX2/RASSF1A methylation panel provides a more balanced diagnostic performance than either biomarker alone and represents a promising adjunctive approach for lung cancer detection. Future studies should focus on standardizing detection methods, integrating these biomarkers with other diagnostic modalities, and evaluating their diagnostic performance in early-stage lung cancer to further enhance their clinical utility.

## 1 Introduction

Lung cancer remains the leading cause of cancer-related deaths worldwide, accounting for over two million new cases and 1.8 million deaths annually (1–3). Despite advancements in treatment strategies, including targeted therapies and immunotherapy, the prognosis for lung cancer, especially in advanced stages, remains dismal, with 5-year survival rates as low as 15% for stage III and 5% for stage IV disease (4–6). The two main types of lung cancer, non-small cell lung cancer (NSCLC) and small cell lung cancer (SCLC) differ in their biological behaviors and therapeutic responses, with NSCLC further classified into subtypes such as squamous cell carcinoma (SCC) and adenocarcinoma (AC) (7, 8).

Early detection of lung cancer is critical for improving survival outcomes, yet most cases are diagnosed at an advanced stage due to the lack of specific symptoms and the limitations of conventional diagnostic methods, such as imaging and invasive biopsies (9–12). In this context, minimally invasive diagnostic tools with higher sensitivity and specificity are urgently needed. Recent advances in the understanding of epigenetics have highlighted the role of DNA methylation as a promising biomarker for cancer detection (13, 14). DNA methylation plays a pivotal role in tumorigenesis, with cancer cells exhibiting either global hypomethylation or specific hypermethylation on promoter regions of tumor suppressor genes (15–17). Promoter hypermethylation is particularly relevant as it frequently silences genes critical for cell differentiation and fate, making it a promising target for biomarker development (18, 19).

To provide background on the development of DNA methylation biomarkers for lung cancer, we summarized 42 previously reported genes, including *RASSF1A, SHOX2, CDKN2A, HOXA1*, and others (Supplementary Material 1), which exhibit aberrant promoter methylation detectable in non-invasive samples such as blood, sputum, and pleural effusion, enabling non-invasive diagnostic approaches (20). Among the most studied methylation biomarkers, *RASSF1A* and *SHOX2* genes have shown significant diagnostic potential (21). Hypermethylation of *RASSF1A* leads to silencing of its tumor-suppressive function, while *SHOX2* methylation has been strongly associated with malignancy (22, 23).

Despite promising findings, the diagnostic efficacy of *RASSF1A* and *SHOX2* methylation remains inconclusive, with reported sensitivity and specificity varying across studies. Therefore, a systematic synthesis of available evidence is needed to evaluate their diagnostic performance comprehensively. This study aims to conduct a systematic review and meta-analysis to assess the diagnostic accuracy of *RASSF1A* and *SHOX2* methylation in lung cancer, focusing on key metrics such as sensitivity, specificity, and diagnostic odds ratio. By analyzing data from 47 studies, this meta-analysis seeks to clarify their utility as biomarkers and guide future research and clinical implementation.

## 2 Materials and methods

### 2.1 Search strategy

To identify relevant studies investigating the diagnostic efficacy of *SHOX2*, *RASSF1A*, and the combination of *SHOX2* and *RASSF1A* methylation in lung cancer, a comprehensive and systematic search was conducted. The databases searched included the Cochrane Library, PubMed, Web of Science, and Embase, covering publications up to July 2026. Medical Subject Headings (MeSH) terms and free-text keywords related to lung cancer and DNA methylation were utilized, including terms such as “lung neoplasms,” “DNA methylation,” “*SHOX2*,” and “*RASSF1A*.” The search strategy was adapted to each database to ensure optimal coverage, focusing on peer-reviewed studies published in English.

### 2.2 Selection criteria

The study selection process adhered to predefined inclusion and exclusion criteria. Human studies employing case-control, cohort, or cross-sectional designs that evaluated the diagnostic performance of SHOX2 methylation, RASSF1A methylation, or their combined methylation panel for lung cancer were included. Eligible studies were required to include both patients with lung cancer and an appropriate control group, provide sufficient data to construct a 2 × 2 contingency table, and be published as original full-text articles.

Studies were excluded if they were conducted on animal models or *in vitro* systems, as these differ significantly in biology and pathology from human lung cancer. Reviews, conference abstracts, editorials, case reports, letters, comments, and non-English publications were also excluded. Studies involving duplicate or overlapping patient populations or those that did not evaluate the diagnostic performance of the biomarkers of interest were also excluded. The study selection process followed the PRISMA (Preferred Reporting Items for Systematic Reviews and Meta-Analyses) guidelines, and the detailed inclusion and exclusion process was summarized using a PRISMA flow diagram.

### 2.3 Data extraction

Eligibility of the identified studies was independently assessed by two reviewers based on the predefined inclusion and exclusion criteria. Discrepancies between the reviewers were resolved through discussion or consultation with a third reviewer. Data extraction was performed independently by three reviewers (T.W., JY.W., and H.X.) using a standardized data collection form. Extracted information included study characteristics, participant demographics, methodological details, and key outcome measures such as sensitivity, specificity, and other diagnostic performance metrics.

For each independent diagnostic dataset, the numbers of true positives (TP), false positives (FP), false negatives (FN), and true negatives (TN) were extracted directly from the original publication whenever available. When these values were not explicitly reported, they were derived from the reported sample sizes and diagnostic accuracy measures only when the corresponding 2 × 2 contingency table could be reconstructed uniquely. If the reported information permitted more than one possible combination of TP, FP, FN, and TN, the data were considered non-reconstructable and were not included in the quantitative synthesis.

Independent cohorts reported within the same publication were treated as separate diagnostic datasets. However, data derived from the same patient population were included only once within each biomarker analysis to avoid double-counting and violation of statistical independence. When multiple diagnostic results based on different sample types or assay methods were reported for the same patient population, only one dataset was retained according to a predefined sample-type hierarchy: tissue > bronchoalveolar lavage fluid > pleural effusion > bronchial aspirate > plasma > serum. This hierarchy prioritized specimens considered to provide a more direct representation of tumor-derived methylation signals.

### 2.4 Quality assessment

The methodological quality and risk of bias of the included studies were independently assessed by two reviewers using the Quality Assessment of Diagnostic Accuracy Studies-2 (QUADAS-2) tool. QUADAS-2 evaluates four key domains: patient selection, index test, reference standard, and flow and timing. Each domain was assessed for risk of bias, while the first three domains were additionally evaluated for concerns regarding applicability. Any discrepancies between the reviewers were resolved through discussion or consultation with a third reviewer. The results of the quality assessment were summarized using standard QUADAS-2 risk-of-bias and applicability judgments to evaluate the overall methodological quality of the included studies.

### 2.5 Meta-analysis

Diagnostic test accuracy meta-analysis was performed to evaluate the diagnostic performance of SHOX2 methylation, RASSF1A methylation, and the combined SHOX2/RASSF1A methylation panel for distinguishing patients with lung cancer from controls.

Pooled diagnostic accuracy was estimated using a bivariate random-effects model (Reitsma model), which jointly models sensitivity and specificity while accounting for their within-study correlation and between-study heterogeneity. Based on the fitted bivariate model, hierarchical summary receiver operating characteristic (HSROC) curves were generated to summarize the overall diagnostic performance of each biomarker panel. Summary estimates of sensitivity, specificity, positive likelihood ratio (PLR), negative likelihood ratio (NLR), diagnostic odds ratio (DOR), and the area under the HSROC curve (AUC) with corresponding 95% confidence intervals (CIs) were calculated.

Potential threshold effects were evaluated by calculating the Spearman correlation coefficient between the logit-transformed sensitivity and the logit-transformed false-positive rate (1 − specificity). A significant positive correlation was considered indicative of a threshold effect. Between-study heterogeneity was explored descriptively using Cochran’s Q and I^2^ statistics for sensitivity, specificity, PLR, NLR, and DOR to facilitate comparison with previous diagnostic meta-analyses, while the primary pooled estimates were obtained from the bivariate random-effects model. Prespecified subgroup analyses were conducted according to sample type, assay method, sample size, ethnicity, and pathological subtype to explore potential sources of heterogeneity. Publication bias was assessed using Deeks’ funnel plot asymmetry test, which is recommended for diagnostic accuracy meta-analyses. Leave-one-out sensitivity analyses were performed to evaluate the robustness of the pooled estimates.

### 2.6 Statistical analysis

All statistical analyses were performed using R (version 4.6.0; R Foundation for Statistical Computing, Vienna, Austria). Diagnostic test accuracy meta-analyses were conducted using the mada package. Pairwise differences in pooled sensitivity and specificity among the combined SHOX2/RASSF1A panel, SHOX2 alone, and RASSF1A alone were evaluated using Wald tests for the corresponding biomarker-group coefficients in the joint bivariate meta-regression model. All statistical tests were two-sided. A P value <0.05 was considered statistically significant.

## 3 Results

### 3.1 Publication search and included studies

**FIGURE 1:**
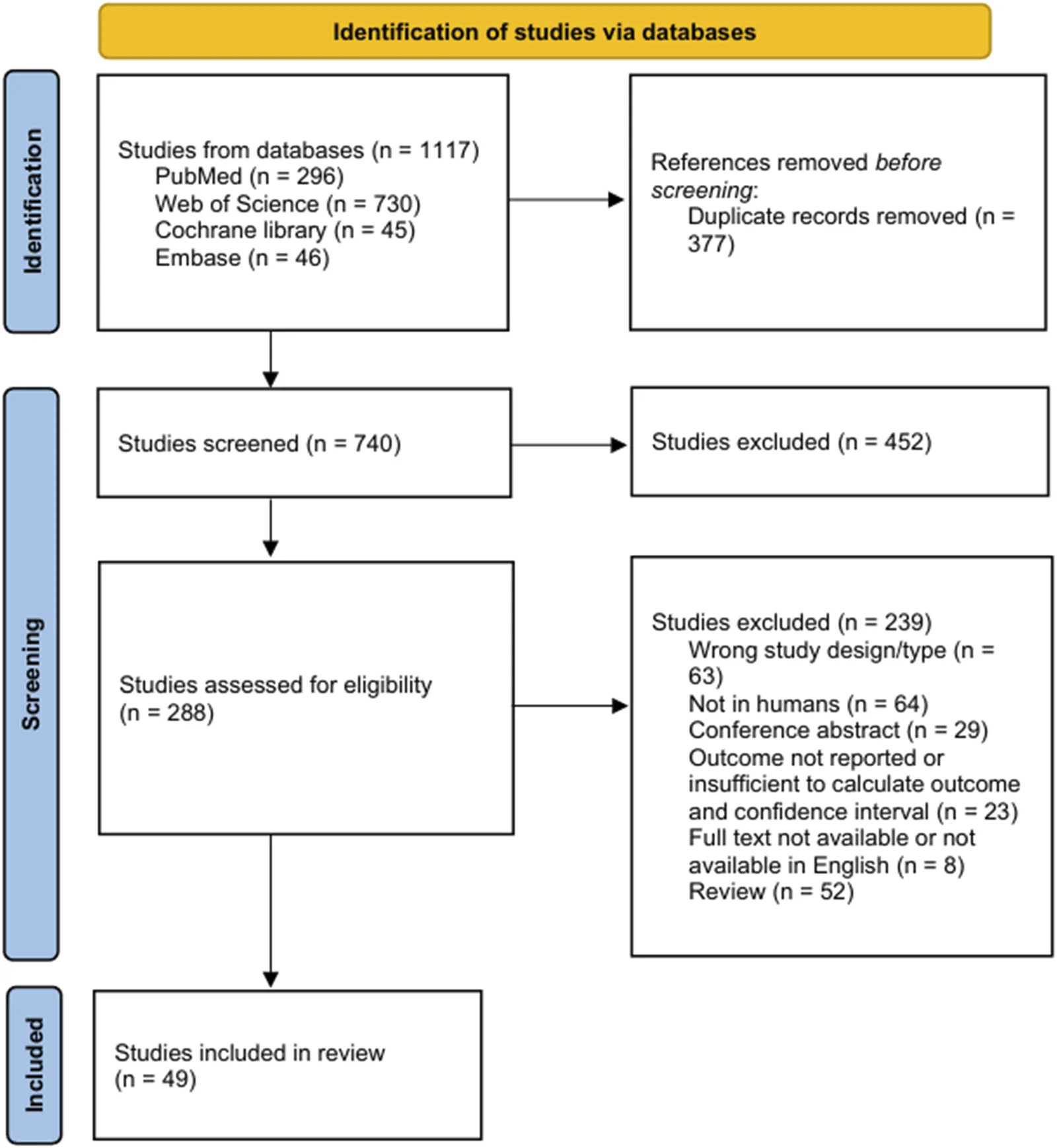
PRISMA flow chart depicting the process for selecting studies in this review.

The study selection process for this meta-analysis is summarized in a PRISMA flow chart (Figure 1). A total of 1117 studies were retrieved from PubMed, Web of Science, Cochrane Library, and Embase. After removing 377 duplicate records, 740 studies were screened. Of these, 452 were excluded based on titles and abstracts, leaving 288 studies for full-text review. Ultimately, 239 studies were excluded for the following reasons: 63 were of incorrect study types, 64 were not conducted on humans, 29 were conference abstracts, 23 lacked sufficient data for outcome calculation, 8 full texts articles were unavailable, and 52 were review articles. Forty-nine studies met the inclusion criteria and were included in this meta-analysis (24–45), (46–72). The QUADAS-2 assessment showed that most studies had a low risk of bias in the flow and timing domain, whereas the patient selection and reference standard domains were predominantly rated as unclear. The index test domain showed the highest proportion of high-risk assessments. Concerns regarding applicability were generally low across all domains. Detailed QUADAS-2 assessments are presented in Supplementary Material 2.

### 3.2 Characteristics of included studies

The detailed characteristics of the 49 included studies are presented in Table 1. These studies, published between 2004 and 2025, included various study designs, participant demographics, sample types, and methodologies. Most studies (45/49) used a case-control design, while four employed a cohort design. The number of cases ranged from 9 to 585, and the number of controls ranged from 9 to 445. Sample types included FFPE tissues, bronchoalveolar lavage fluid (BALF), pleural effusion, bronchial epithelial cells, blood, bronchial aspirates, lung tissues, bronchial washings, and sputum. Because some studies evaluated multiple specimen types, the summed frequencies across specimen types exceeded the total number of included studies.

**TABLE 1:** Summary of included study characteristics.

Study ID	Country	Genes	Assay method	Study design	Cases	Controls	Cohort	Sample	Number of cases	Number of controls	Histology	Stage	Reference standard
Zhao (57)	China	SHOX2, RASSF1A	MSP	Case-control study	Retrospectively selected cases	Combination of benign diseases and AIS	Training	FFPE	207	65	AIS 31/238 MIA 36/238 IA 171/238	T0 31/238 IA 186/238 IB 11/238 II ∼ IV 10/238	Histopathology
Chen (55)	China	SHOX2, RASSF1A	MSP	Case-control study	Retrospectively selected cases	Matched non-tumor lung tissue specimens	Training	Lung tissues	25	25	LUAD 25/50	I 2250 II 3/50	Histopathology
Zeng (53)	China	SHOX2, RASSF1A	MSP	Case-control study	Retrospectively selected cases	Not described	Training	BALF	57	20	Not reported	Not reported	Histopathology
Gu (54)	China	SHOX2, RASSF1A	RT-PCR	Case-control study	Retrospectively selected cases	Combination of benign diseases	Training	Lung tissues	65	29	LUSC 19/94 LUAD 28/94 SCLC 8/94	I/II 7/94 III 19/94 IV 39/94	Histopathology
​	​	​	​	​	​	​	Training	Lymph node	62	17	LUSC 7/79 LUAD 39/79 SCLC 10/79	I/II 1/79 III 18/79 IV 43/79	Histopathology
Ren (63)	China	SHOX2, RASSF1A	qPCR	Case-control study	Retrospectively selected cases	Combination of benign diseases and other cancers	Training	BALF	123	130	LUSC 17/123 LUAD 82/123 SCLC 8/123 Others 16/123	T0 4/123 I 47/123 II 13/123 III 19/123 IV 25/123 Unknown 15/123	Surgery specimen/histopathology
Zhong (56)	China	SHOX2, RASSF1A	MSP	Case-control study	Retrospectively selected cases	BPE, not further described	Training	PE	68	110	LUSC 9/68 LUAD 51/68 Others 8/68	Not reported	Not described
Zhang (58)	China	SHOX2, RASSF1A	qPCR	Case-control study	Retrospectively selected cases	Combination of benign diseases	Training	PE	45	45	NSCLC 42/45 SCLC 3/45	Not reported	Pleural tissue biopsy/cytology
Zhang (59)	China	SHOX2, RASSF1A	QMSP	Case-control study	Retrospectively selected cases	Combination of benign diseases and AAH	Training	BALF	585	101	LUSC 162/585 LUAD 181/585 SCLC 135/585 Others 107/585	Not reported	Clinical diagnosis
Zhang (60)	China	SHOX2, RASSF1A	RT-PCR	Case-control study	Retrospectively selected cases	Combination of benign diseases and other cancers	Training	BALF	284	38	LUSC 107/284 LUAD 92/284 SCLC 42/284 Others 43/284	I 28/284 II 30/284 III 133/284 IV 93/284	Histopathology/cytology
Shi (62)	China	SHOX2, RASSF1A	QMSP	Case-control study	Retrospectively selected cases	Combination of benign diseases	Training	FFPE	137	114	LUSC 51/137 LUAD 70/137 SCLC 16/137	I 57/137 II 23/137 III 30/137 IV 27/137	Histopathology
Lu (64)	China	SHOX2, RASSF1A	qPCR	Case-control study	Retrospectively selected cases	Combination of benign diseases	Training	BALF	43	23	LUSC 23/43 LUAD 13/43 SCLC 4/43 Others 3/43	I/II 11/43 III/IV 29/43 Unknown 3/43	Histopathology
Liu (65)	China	SHOX2, RASSF1A	QMSP	Case-control study	Retrospectively selected cases	Combination of benign diseases	Training	BEC	185	55	LUSC 53/185 LUAD 104/185 SCLC 23/185 Others 5/185	I 28/185 II 20/185 III 40/185 IV 97/185	Not described
Liang (66)	China	SHOX2, RASSF1A	MSP	Cohort study	Prospectively selected cases	Non-cancer participants who underwent diagnostic work-up	Training	PE	68	48	LUSC 6/68 LUAD 54/68 SCLC 4/68 Others 4/68	Not reported	Histopathology/cytology
Jin (67)	China	SHOX2, RASSF1A	qPCR	Case-control study	Retrospectively selected cases	Non-cancer, not further described	Training	BALF	36	19	MIA 22/36 IA 8/36 others 6/36	Tis 4/36 T1mi/T1a 23/36 T1b 6/36 T1c 3/36	Not described
​	​	​	​	​	​	​	​	Plasma	36	19	MIA 22/36 IA 8/36 others 6/36	Tis 4/36 T1mi/T1a 23/36 T1b 6/36 T1c 3/36	​
Gao (69)	China	SHOX2, RASSF1A	qPCR	Cohort study	Diagnostic work-up for LC	Non-cancer participants who underwent diagnostic work-up	Training	FFPE (tumer)	54	31	AIS 3/54 MIA 10/54 IA 41/54	Tis 3/54 IA 36/54 IB 8/54 II 7/5	Histopathology
​	​	​	​	​	​	​	​	FFPE (tumer and paracancerous)	54	31	AIS 3/54 MIA 10/54 IA 41/54	Tis 3/54 IA 36/54 IB 8/54 II 7/54	​
Chen (70)	China	SHOX2, RASSF1A	qPCR	Case-control study	Retrospectively selected cases	Combination of benign diseases	Training	PE	35	33	LUSC 1/35 LUAD 23/35 SCLC 1/35 Others 10/35	Not reported	Histopathology/cytology
Xiang (68)	China	SHOX2, RASSF1A	MSP	Case-control study	Retrospectively selected cases	Combination of AIS and AAH	Training	FFPE	133	125	MIA 59/133 IA 74/133	Not reported	Histopathology
Xie (61)	China	SHOX2, RASSF1A	RT-PCR	Case-control study	Retrospectively selected cases	Combination of benign diseases	Training	BALF	72	35	LUSC 23/72 LUAD 35/72 SCLC 14/72	I 22/72 II 27/72 III 13/72 IV 10/72	Histopathology
Xu (48)	China	SHOX2	qPCR	Case-control study	Retrospectively selected cases	Unmatched healthy controls	Training	Blood	302	153	LUSC 28/302 LUAD 236/302 SCLC 32/302 Others 6/302	I 68/302 II 62/302 III 72/302 IV 100/302	Tumor tissue biopsy/histopathology
Vo (49)	Vietnam	SHOX2	qPCR	Case-control study	Retrospectively selected cases	Combination of benign diseases	Training	FFPE	120	94	LUAD	III 52/120 IV 59/120 Unknown 9/120	Not described
​	​	​	​	​	​	Unmatched healthy controls	​	Plasma	30	27	Not reported	I 2/30 II 8/30 III 15/30 IV 5/30	Not described
Schmidt (50)	Germany,UK	SHOX2	qPCR	Case-control study	Retrospectively selected cases	Non-cancer, not further described	Training	Bronchial aspirates	281	242	LUSC 103/281 LUAD 109/281 SCLC 29/281 Others 40/281	I 59/281 II 43/281 III 108/281 IV 62/281 Unknown 9/281	Histopathology/cytology
Kneip (51)	Germany,USA	SHOX2	qPCR	Case-control study	Retrospectively selected cases	Combination of healthy, benign diseases and other cancers	Testing	Plasma	188	155	LUSC 38/188 LUAD 31/188 SCLC 15/188 Others 104/188	I 37/188 II 29/188 III 53/188 IV 42/188 Unknown 27/188	Not described
​	​	​	​	​	​	Unmatched healthy controls	Training	Plasma	20	20	Not reported	IV	​
Huang (52)	China	SHOX2	qPCR	Case-control study	Retrospectively selected cases	Combination of benign diseases	Training	Plasma	104	36	LUSC 15/104 LUAD 53/104 SCLC 3/104 Others 33/104	I 48/104 II 15/104 III 20/104 IV 21/104	Histopathology
​	​	​	​	​	​	​	Validation	​	19	11	LUSC 4/19 LUAD 14/19 Others 1/19	I 12/19 II 4/19 III 3/19	​
Feng (71)	China	SHOX2	MSP	Case-control study	Retrospectively selected cases	Combination of benign diseases	Training	Lung tissues	89	9	LUSC 34/89 LUAD 47/89 Others 8/89	I/II 52/89 III/IV 37/89	Surgery specimen/histopathology
Dietrich (72)	UK	SHOX2	qPCR	Case-control study	Retrospectively selected cases	Not described	Validation	BALF	100	104	LUSC 28/125 LUAD 26/125 SCLC 40/125 Others 31/125	Not reported	Histopathology/cytology
Yu (24)	China	RASSF1A	MSP	Case-control study	Retrospectively selected cases	Combination of healthy and benign diseases	Training	Serum	75	50	LUSC 26/75 LUAD 40/75 Others 9/75	I 9/75 II 18/75 IIIa 15/75 IIIb 13/75 IV 2/75	Not described
Shivapurkar (27)	USA	RASSF1A	qPCR	Case-control study	Retrospectively selected cases	Unmatched healthy controls	Training	Lung tissues	40	40	LUSC 18/40 LUAD 22/40	Not reported	Tumor tissue biopsy/histopathology
Smetannikova (26)	Russia	RASSF1A	GLAD-PCR	Case-control study	Retrospectively selected cases	Unmatched healthy controls	Training	Lung tissues	40	25	LUSC 19/40 LUAD 13/40 SCLC 2/40 Others 6/40	I 5/188 II 12/40 III 18/40 IV 4/40 Unknown 1/40	Tumor tissue biopsy/histopathology
Shah (28)	India	RASSF1A	MSP	Case-control study	Prospectively selected cases	Matched on certain characteristics of healthy controls	Training	Blood	100	100	LUSC 28/100 LUAD 72/100	I/II 43/100 III/IV 57/100	Not described
Wang (25)	China	RASSF1A	MSP	Case-control study	Prospectively selected cases	Combination of healthy and benign diseases	Training	Blood	80	50	LUSC 26/80 LUAD 40/80 SCLC 5/80 Others 9/80	I/II 27/80 III/IV 53/80	Histopathology/cytology
Schmiemann (29)	Germany	RASSF1A	QMSP	Cohort study	Retrospectively selected cases	Non-cancer participants who underwent diagnostic work-up	Training	Bronchial aspirates	85	102	LUSC 16/85 LUAD 33/85 SCLC 17/85 Others 19/80	Not reported	Histopathology/cytology
Rykova (30)	Russia	RASSF1A	MSP	Case-control study	Retrospectively selected cases	Combination of benign diseases	Training	Plasma	9	16	Not reported	Not reported	Not described
Roncarati (31)	Italy	RASSF1A	dd-PCR	Cohort study	Diagnostic work-up for LC	Non-cancer participants who underwent diagnostic work-up	Training	BW	91	31	LUSC 32/91 LUAD 41/91 SCLC 11/91 Others 7/91	I 13/91 II 7/91 III 25/91 IV 43/91 Unknown 3/91	Histopathology/cytology
Ponomaryova (32)	Russia	RASSF1A	MSP	Case-control study	Retrospectively selected cases	Matched on certain characteristics of healthy controls	Training	Blood	60	32	LUSC 40/60 LUAD 20/60	I/II 20/60 III 40/60	Histopathology
​	​	​	​	​	​	Matched on certain characteristics of healthy controls	​	Blood	60	32	LUSC 40/60 LUAD 20/60	I/II 20/60 III 40/60	​
Peng (33)	China	RASSF1A	MSP	Case-control study	Retrospectively selected cases	Combination of benign diseases	Training	Sputum	82	25	LUSC 38/82 LUAD 27/82 SCLC 7/82 Others 10/82	I 6/82 II 18/82 III 41/82 IV 17/82	Histopathology
Lewandoska (34)	Poland	RASSF1A	qPCR,MSP	Case-control study	Retrospectively selected cases	Surrounding normal lung tissues from the same cases	Training	Lung tissues	59	59	LUSC 34/59 LUAD 20/59 Others 5/59	I 11/59 II 21/59 III 27/59	Not described
Nunes (35)	Portugal	RASSF1A	QMSP	Case-control study	Retrospectively selected cases	Combination of benign diseases	Training	Plasma	129	28	LUSC 42/129 LUAD 65/129 SCLC 19/129 Others 3/129	I 15/129 II 11/129 III 27/129 IV 46/129	Histopathology/cytology
Nawaz (36)	China	RASSF1A	MMSP	Case-control study	Retrospectively selected cases	Combination of benign diseases	Training	Lung tissues	70	24	NSCLE	Not reported	Not described
Mohammed (37)	Iraq	RASSF1A	MSP	Case-control study	Retrospectively selected cases	Unmatched healthy controls	Training	Sputum	84	42	Not reported	I 11/84 II 20/84 III 35/84 IV 18/84	Histopathology/cytology
Ma (38)	China	RASSF1A	PCR	Case-control study	Retrospectively selected cases	Surrounding normal lung tissues from the same cases	Training	Lung tissues	50	50	LUSC 25/50 LUAD 25/50	I 25/50 II 25/50	Histopathology/cytology
​	​	​	​	​	​	Unmatched healthy controls	​	Bronchial aspirates	40	10	LUSC 16/40 LUAD 24/40	I 23/40 II 17/40	​
Liu (39)	China	RASSF1A	MSP	Case-control study	Retrospectively selected cases	Combination of healthy and benign diseases	Training	Plasma	96	32	Not reported	Not reported	Not described
​	​	​	​	​	​	​	​	Lung tissues	96	32	Not reported	Not reported	​
Kim (40)	Korea	RASSF1A	MSP	Case-control study	Retrospectively selected cases	Non-cancer, not further described	Training	Lung tissues	85	127	LUSC 43/85 LUAD 31/85 Others 11/85	I 52/85 II 33/85	Histopathology/cytology
​	​	​	​	​	​	​	​	BALF	85	127	LUSC 43/85 LUAD 31/85 Others 11/85	I 52/85 II 33/85	​
Hubers (41)	Netherlands	RASSF1A	MSP	Case-control study	Retrospectively selected cases	Combination of benign diseases	Training	Sputum	98	90	Not reported	Not reported	Histopathology/cytology
​	​	​	​	​	​	​	Validation	​	60	445	Not reported	Not reported	​
Hubers (43)	Netherlands	RASSF1A	MSP	Case-control study	Prospectively selected cases	Non-cancer, not further described	Training	Sputum	56	217	LUSC 7/56 LUAD 34/56 SCLC 2/56 Others 13/56	I 36/56 II 4/56 III 6/56 IV 10/56	Histopathology/cytology
Hubers (44)	Netherlands	RASSF1A	MSP	Case-control study	Retrospectively selected cases	Combination of benign diseases	Learning	Sputum	73	86	LUSC 31/73 LUAD 26/73 SCLC 1/73 Others 15/73	I 14/73 II 9/73 III 24/73 IV 25/73 Unknown 1/73	Histopathology/cytology
​	​	​	​	​	​	​	Validation	​	159	154	LUSC 50/159 LUAD 66/159 SCLC 6/159 Others 37/159	I 29/159 II 17/159 III 47/159 IV 66/159	​
Grote (45)	Germany	RASSF1A	QMSP	Case-control study	Retrospectively selected cases	Combination of benign diseases	Training	Bronchial aspirates	157	46	LUSC 48/157 LUAD 42/157 SCLC 40/157 Others 27/157	Not reported	Histopathology/cytology
Gao (46)	China	RASSF1A	QMSP	Case-control study	Retrospectively selected cases	Combination of healthy and benign diseases	Training	Plasma	58	54	LUSC 23/58 LUAD 18/58 SCLC 2/58 Others 15/58	Not reported	Histopathology/cytology
​	​	​	​	​	​	​	​	Sputum	40	36	LUSC 13/40 LUAD 13/40 SCLC 2/40 Others 12/40	Not reported	​
​	​	​	​	​	​	​	​	Lung tissues	39	15	LUSC 18/39 LUAD 12/39 SCLC 2/39 Others 7/39	Not reported	​
Constâncio (47)	Portugal	RASSF1A	MSP	Case-control study	Retrospectively selected cases	Asymptomatic controls, not further described	Training	Plasma	102	136	LUSC 42/102 LUAD 43/102 SCLC 16/102 Others 1/102	I/II 17/102 III/IV 85/102	Histopathology/cytology
Hubers (42)	Netherlands	RASSF1A	QMSP	Case-control study	Prospectively selected cases	Combination of benign diseases	Training	Sputum1-3	53	47	Not reported	Not reported	Histopathology/cytology
​	​	​	​	​	​	​	​	Sputum4-6	53	47	Not reported	Not reported	​
​	​	​	​	​	​	​	​	Sputum7-9	53	47	Not reported	Not reported	​

### 3.3 Meta-analysis results of *SHOX2 and RASSF1A* methylation

**FIGURE 2:**
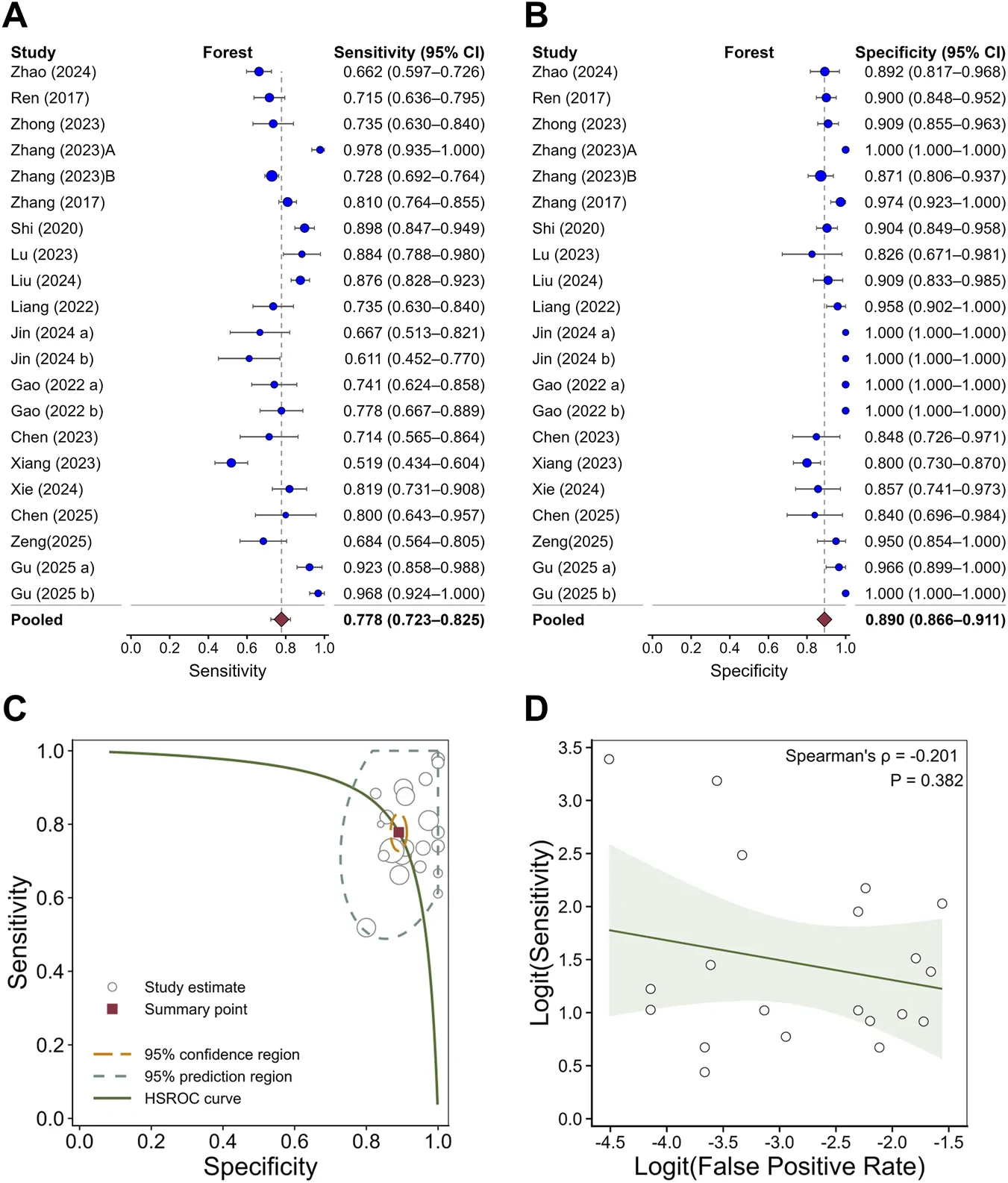
Diagnostic performance of the combined SHOX2/RASSF1A methylation panel for lung cancer detection. **(A)** Forest plot of pooled sensitivity. **(B)** Forest plot of pooled specificity. **(C)** Hierarchical summary receiver operating characteristic curve showing the summary operating point, 95% confidence region, and 95% prediction region. **(D)** Assessment of the threshold effect using Spearman correlation analysis between the logit-transformed sensitivity and false-positive rate.

The combined diagnostic performance of *SHOX2* and *RASSF1A* methylation for lung cancer is shown in Figure 2. Based on 18 studies (53–70), the pooled sensitivity was 0.778 (0.723–0.825), the pooled specificity was 0.890 (0.866–0.911), the PLR was 7.102 (5.565–9.063), the NLR was 0.249 (0.196–0.318), the DOR was 28.483 (18.095–44.835), and the area under the HSROC curve was 0.916 (0.872–0.924). Forest plots of pooled sensitivity and specificity are presented in Figures 2A,B, respectively, while the HSROC curve is shown in Figure 2C. Spearman correlation analysis demonstrated no significant threshold effect among the included studies (Spearman’s ρ = −0.201, P = 0.382; Figure 2D), suggesting that heterogeneity was unlikely to be attributable to differences in diagnostic thresholds.

### 3.4 Meta-analysis results of *SHOX2* methylation

**FIGURE 3:**
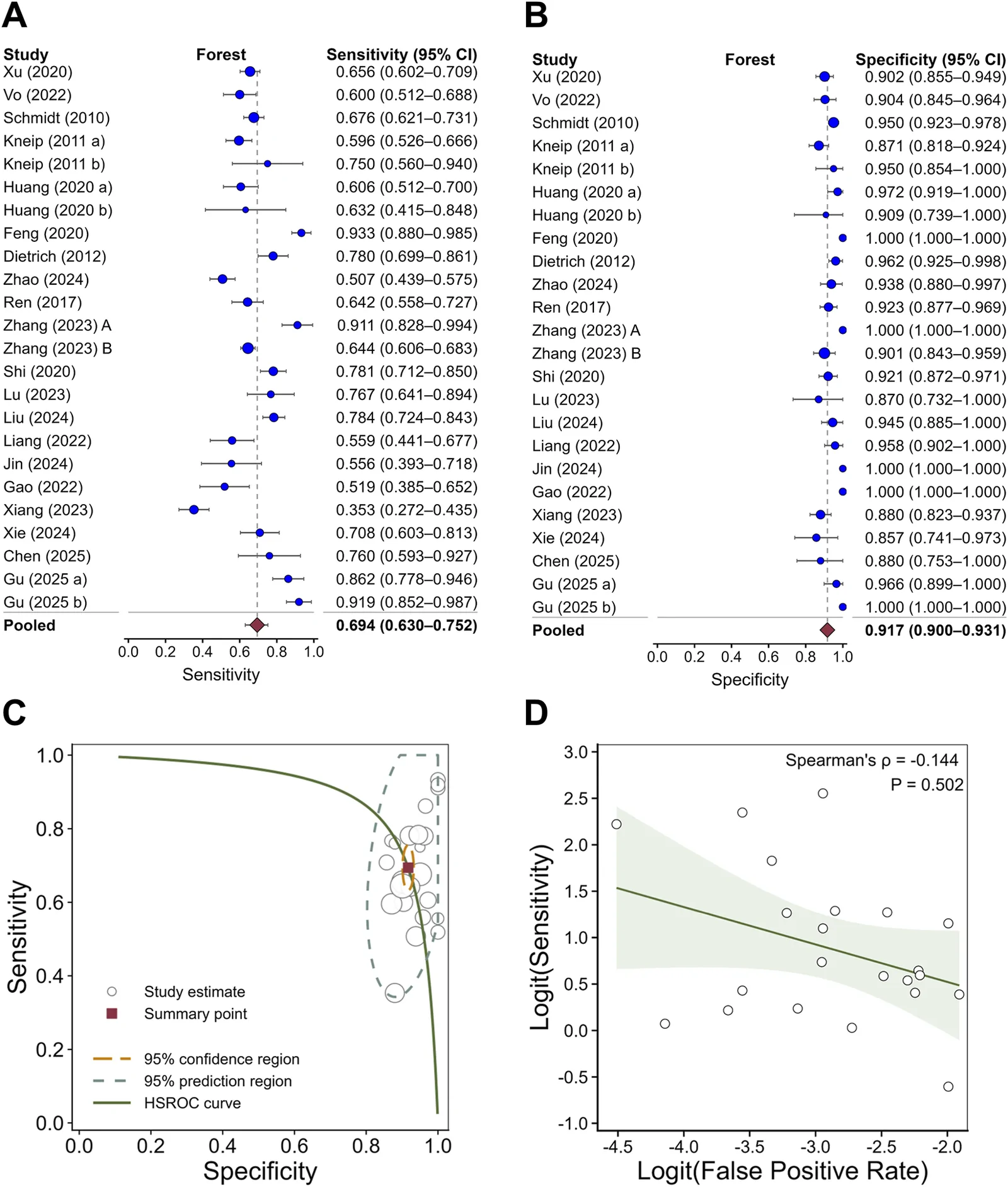
Diagnostic performance of SHOX2 methylation for lung cancer detection. **(A)** Forest plot of pooled sensitivity. **(B)** Forest plot of pooled specificity. **(C)** Hierarchical summary receiver operating characteristic (HSROC) curve showing the summary operating point, 95% confidence region, and 95% prediction region. **(D)** Assessment of the threshold effect using Spearman correlation analysis between the logit-transformed sensitivity and false-positive rate. HSROC, hierarchical summary receiver operating characteristic.

The diagnostic performance of *SHOX2* methylation was evaluated in 21 studies (48–52, 54, 55, 57–59, 61–69, 71, 72). The pooled sensitivity was 0.694 (0.630–0.752), the pooled specificity was 0.917 (0.900–0.931), the PLR was 8.363 (6.584–10.623), the NLR was 0.334 (0.271–0.411), the DOR was 25.071 (16.468–38.166), and the area under the HSROC curve was 0.925 (0.890–0.930). The corresponding forest plots and HSROC curve are presented in Figures 3A–C. No significant threshold effect was identified (Spearman’s ρ = −0.144, P = 0.502; Figure 3D).

### 3.5 Meta-analysis results of *RASSF1A* methylation

**FIGURE 4:**
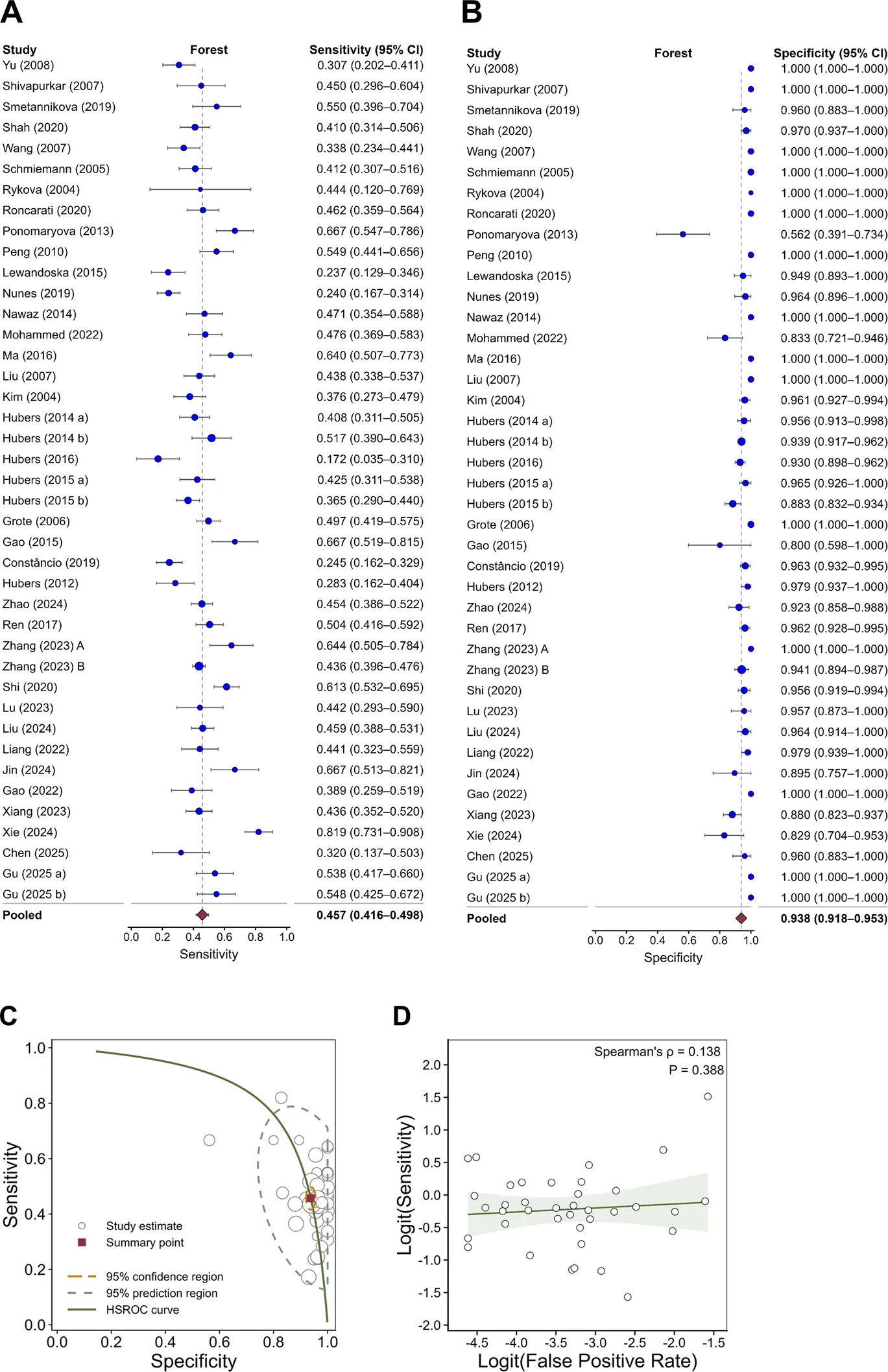
Diagnostic performance of RASSF1A methylation for lung cancer detection. **(A)** Forest plot of pooled sensitivity. **(B)** Forest plot of pooled specificity. **(C)** Hierarchical summary receiver operating characteristic (HSROC) curve showing the summary operating point, 95% confidence region, and 95% prediction region. **(D)** Assessment of the threshold effect using Spearman correlation analysis between the logit-transformed sensitivity and false-positive rate. HSROC, hierarchical summary receiver operating characteristic.

The diagnostic performance of *RASSF1A* methylation was evaluated in 38 studies (24–47, 54, 55, 57–59, 61–69). The pooled sensitivity was 0.457 (0.416–0.498), the pooled specificity was 0.938 (0.918–0.953), the PLR was 7.338 (5.624–9.573), the NLR was 0.579 (0.540–0.622), the DOR was 12.666 (9.474–16.934), and the area under the HSROC curve was 0.789 (0.721–0.868). Figures 4A–C presents the corresponding forest plots and HSROC curve. No significant threshold effect was observed (Spearman’s ρ = 0.138, P = 0.388; Figure 4D).

### 3.6 Pairwise comparison of diagnostic performance

Compared with SHOX2 alone, the combined SHOX2/RASSF1A assay showed higher sensitivity (P = 0.019), whereas specificity did not differ significantly (P = 0.272). Compared with RASSF1A alone, the combined assay showed higher sensitivity (P < 0.001) and lower specificity (P = 0.015). SHOX2 also showed higher sensitivity than RASSF1A (P < 0.001), while their specificities did not differ significantly (P = 0.183) (Table 2).

**TABLE 2:** Pairwise comparisons of diagnostic performance among biomarkers via bivariate meta-regression.

Comparison	P value for sensitivity	P value for specificity
SHOX2&RASSF1A vs. SHOX2	0.019	0.272
RASSF1A vs. SHOX2	<0.001	0.183
SHOX2&RASSF1A vs. RASSF1A	<0.001	0.015

### 3.7 Evaluation of heterogeneity and publication bias

**FIGURE 5:**
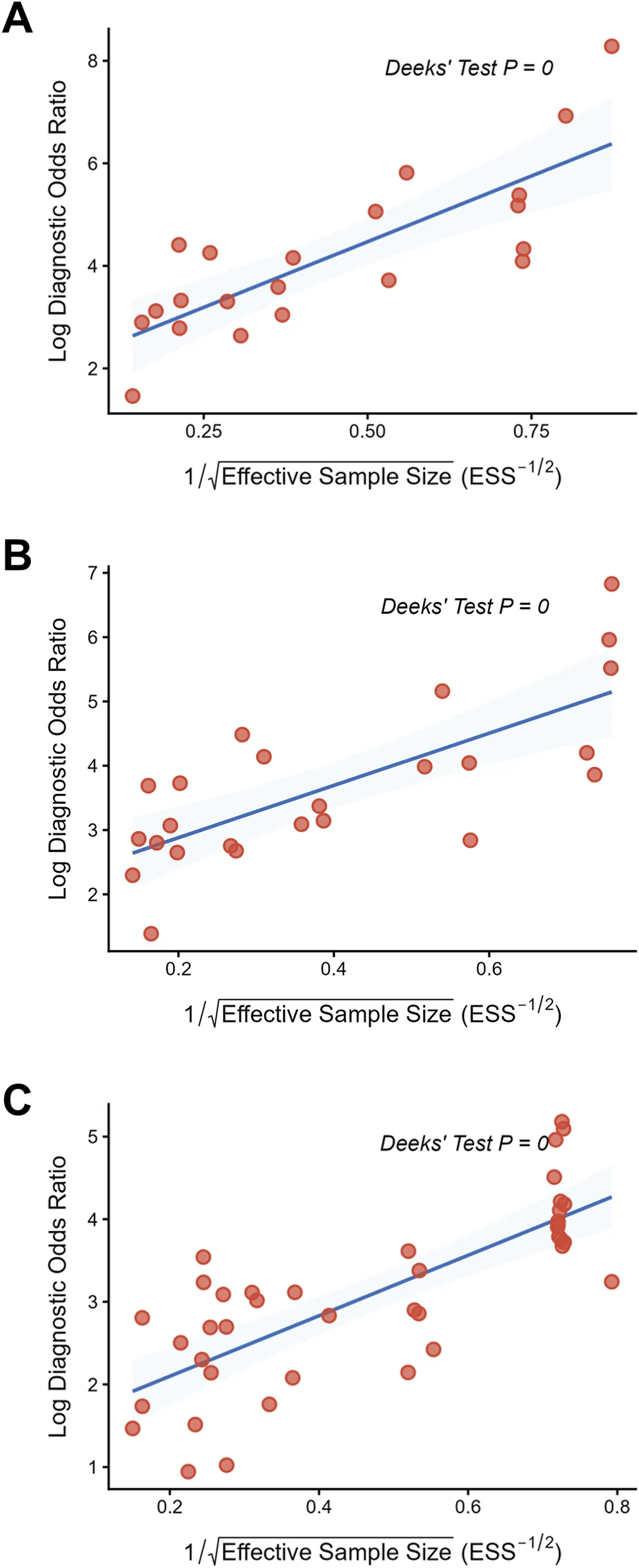
Assessment of publication bias using Deeks’ funnel plot asymmetry test. **(A)** Combined SHOX2/RASSF1A methylation panel. **(B)** SHOX2 methylation. **(C)** RASSF1A methylation. Publication bias was evaluated by regressing the log diagnostic odds ratio against the inverse root of the effective sample size (ESS^−1/2). A significant non-zero slope indicates potential publication bias.

Heterogeneity statistics are summarized in Supplementary Table S1. Significant heterogeneity was observed for pooled sensitivity in the combined SHOX2/RASSF1A, SHOX2, and RASSF1A analyses (all P < 0.001; I^2^ = 87.29%, 91.96%, and 83.32%, respectively). In contrast, specificity showed low heterogeneity for the combined SHOX2/RASSF1A (I^2^ = 26.98%, P = 0.063) and SHOX2 analyses (I^2^ = 19.14%, P = 0.312), whereas moderate heterogeneity was observed for RASSF1A (I^2^ = 66.69%, P < 0.001). Deeks’ funnel plot asymmetry test demonstrated significant publication bias for the combined SHOX2/RASSF1A, SHOX2, and RASSF1A analyses (all P < 0.001; Figures 5A–C).

### 3.8 Subgroup analysis

To further explore potential sources of between-study heterogeneity, prespecified subgroup analyses were performed according to ethnicity, sample type, sample size, assay method, and pathological subtype. For the combined SHOX2/RASSF1A analysis, no significant differences in pooled sensitivity or specificity were observed across sample types or sample size categories (all P > 0.05) (Table 3). However, pooled sensitivity differed significantly according to assay method (P = 0.005), with QMSP and RT-PCR showing higher sensitivities than MSP, whereas specificity did not differ significantly among assay methods (P = 0.730). Studies including multiple pathological subtypes also showed higher sensitivity than those restricted to a single subtype (P = 0.001), with a significant difference in specificity (P = 0.028). For SHOX2 (Table 4), pooled sensitivity varied significantly according to sample type (P = 0.001) and pathological subtype (P < 0.001), whereas no significant subgroup differences were observed for ethnicity, sample size, assay method, or specificity (all P > 0.05). For RASSF1A (Table 5), assay method significantly influenced pooled sensitivity (P = 0.043), while no significant subgroup differences were identified for ethnicity, sample type, sample size, pathological subtype, or specificity (all P > 0.05), although ethnicity showed a borderline association with sensitivity (P = 0.052).

**TABLE 3:** The sensitivity and specificity subgroup analyses of SHOX2 and RASSF1A: Sample types, sample size, assay methods, and pathological types.

Parameter	Subgroup	No. of studies	Pooled sensitivity (95% CI)	*P* for subgroup difference	Pooled specificity (95% CI)	*P* for subgroup difference
Sample type	​	​	​	​	​	​
​	Liquid	​	​	​	​	​
​	BALF	7	0.76 (0.71, 0.80)	0.237	0.89 (0.85, 0.92)	0.386
​	PE	4	0.75 (0.68–0.80)	​	0.91 (0.85–0.95)	​
​	BEC	1	0.88 (0.82–0.92)	​	0.91 (0.80–0.96)	​
​	Plasma	1	0.61 (0.45–0.75)	​	0.97 (0.70–1.00)	​
​	Tissue	​	​	​	​	​
​	FFPE	4	0.73 (0.53–0.86)	​	0.88 (0.80–0.93)	​
Sample size	​	​	​	​	​	​
​	<100	7	0.76 (0.68, 0.83)	0.823	0.88 (0.81, 0.93)	0.981
​	≥100	10	0.76 (0.69–0.82)	​	0.89 (0.85–0.91)	​
Assay method	​	​	​	​	​	​
​	MSP	6	0.68 (0.59, 0.75)	**0.005**	0.88 (0.82, 0.93)	0.730
​	qPCR	6	0.76 (0.68–0.83)	​	0.89 (0.85–0.93)	​
​	QMSP	3	0.84 (0.72–0.92)	​	0.89 (0.85–0.92)	​
​	RT-PCR	2	0.81 (0.77–0.85)	​	0.92 (0.68–0.99)	​
Multifocality	​	​	​	​	​	​
​	Solitary	4	0.64 (0.54–0.73)	**0.001**	0.90 (0.76–0.96)	**0.028**
​	Multiple	11	0.81 (0.75–0.85)	​	0.90 (0.87–0.92)	​

BALF, bronchoalveolar lavage fluid; PE, pleural effusion; BEC, bronchial epithelial cells; FFPE, formalin-fixed paraffin-embedded; MSP, Methylation-Specific Polymerase Chain Reaction; qPCR, Quantitative Polymerase Chain Reaction; QMSP, Quantitative Methylation-Specific Polymerase Chain Reaction; RT-PCR, Reverse Transcription Polymerase Chain Reaction.

Bold values indicate statistical significance (P < 0.05).

**TABLE 4:** The sensitivity and specificity subgroup analyses of SHOX2: Different races, sample types, sample size, assay methods, and pathological types.

Parameter	Subgroup	No. of studies	Pooled sensitivity (95% CI)	*P* for subgroup difference	Pooled specificity (95% CI)	*P* for subgroup difference
Ethnicity	Asian	19	0.68 (0.61, 0.75)	0.832	0.91 (0.89, 0.93)	0.526
​	White	4	0.69 (0.60–0.77)	​	0.93 (0.87–0.97)	​
Sample type	​	​	​	​	​	​
​	Liquid	​	​	​	​	​
​	Blood	1	0.66 (0.60–0.71)	**0.001**	0.90 (0.84–0.94)	0.186
​	Plasma	6	0.63 (0.54–0.71)	​	0.92 (0.85–0.96)	​
​	BALF	6	0.68 (0.62–0.74)	​	0.91 (0.88–0.94)	​
​	PE	2	0.77 (0.31–0.96)	​	0.97 (0.90–0.99)	​
​	BA	1	0.68 (0.62–0.73)	​	0.95 (0.91–0.97)	​
​	BEC	1	0.78 (0.72–0.84)	​	0.95 (0.84–0.98)	​
​	Tissue	​	​	​	​	​
​	FFPE	5	0.56 (0.41–0.70)	​	0.91 (0.87–0.93)	​
​	Lung tissues	2	0.90 (0.80, 0.95)	​	0.96 (0.83, 0.99)	​
Sample size	<100	12	0.74 (0.63, 0.82)	0.096	0.94 (0.89, 0.96)	0.313
​	≥100	14	0.64 (0.58–0.70)	​	0.92 (0.90–0.93)	​
Assay method	qPCR	14	0.67 (0.62–0.72)	0.390	0.92 (0.90–0.94)	0.840
​	MSP	4	0.63 (0.31–0.87)	​	0.92 (0.86–0.96)	​
​	QMSP	3	0.74 (0.63–0.82)	​	0.92 (0.88–0.94)	​
​	RT-PCR	2	0.79 (0.60, 0.90)	​	0.91 (0.71, 0.98)	​
Tumor focality	Solitary	5	0.50 (0.41–0.59)	**<0.001**	0.90 (0.87–0.93)	0.791
​	Multiple	15	0.72 (0.65–0.77)	​	0.92 (0.90–0.94)	​
​	Unknown	2	0.80 (0.66–0.89)	​	0.94 (0.82–0.98)	​

BALF, bronchoalveolar lavage fluid; BA, Bronchial aspirates; PE, pleural effusion; BEC, bronchial epithelial cells; FFPE: formalin-fixed paraffin-embedded; qPCR, Quantitative Polymerase Chain Reaction; MSP, Methylation-Specific Polymerase Chain Reaction; QMSP, Quantitative Methylation-Specific Polymerase Chain Reaction; RT-PCR, Reverse Transcription Polymerase Chain Reaction.

Bold values indicate statistical significance (P < 0.05).

**TABLE 5:** The sensitivity and specificity subgroup analyses of RASSF1A: Different races, sample types, sample size, assay methods, and pathological types.

Parameter	Subgroup	No. of studies	Pooled sensitivity (95% CI)	*P* for subgroup difference	Pooled specificity (95% CI)	*P* for subgroup difference
Ethnicity	​	​	​	​	​	​
​	Asian	23	0.48 (0.43, 0.53)	0.052	0.95 (0.93, 0.97)	0.213
​	White	17	0.40 (0.34–0.46)	​	0.94 (0.90–0.97)	​
Sample type	​	​	​	​	​	​
​	Liquid	​	​	​	​	​
​	Serum	1	0.31 (0.21–0.42)	0.453	0.99 (0.86–1.00)	0.071
​	Blood	3	0.47 (0.28–0.66)	​	0.93 (0.49–0.99)	​
​	BALF	6	0.45 (0.27–0.64)	​	0.94 (0.89–0.97)	​
​	BA	3	0.49 (0.40–0.58)	​	0.98 (0.86–1.00)	​
​	Plasma	6	0.33 (0.24–0.42)	​	0.97 (0.94–0.98)	​
​	BW	1	0.46 (0.36–0.56)	​	0.98 (0.79–1.00)	​
​	Sputum	9	0.42 (0.36–0.49)	​	0.93 (0.90–0.96)	​
​	PE	2	0.54 (0.34–0.73)	​	0.98 (0.92–1.00)	​
​	BEC	1	0.46 (0.39–0.53)	​	0.96 (0.87–0.99)	​
​	Tissue	​	​	​	​	​
​	Lung tissues	10	0.47 (0.39, 0.56)	​	0.97 (0.94, 0.98)	​
​	FFPE	4	0.48 (0.38–0.57)	​	0.93 (0.87–0.96)	​
Sample size	​	​	​	​	​	​
​	<100	13	0.51 (0.44, 0.57)	0.077	0.94 (0.90–0.97)	0.655
​	≥100	29	0.43 (0.38–0.48)	​	0.95 (0.93–0.96)	​
Assay method	​	​	​	​	​	​
​	MSP	18	0.42 (0.37–0.46)	**0.043**	0.94 (0.90–0.96)	0.270
​	qPCR	7	0.42 (0.33–0.53)	​	0.96 (0.94–0.98)	​
​	GLAD-PCR	1	0.55 (0.40–0.69)	​	0.96 (0.76–0.99)	​
​	QMSP	8	0.45 (0.35–0.55)	​	0.96 (0.94–0.98)	​
​	ddPCR	1	0.46 (0.36–0.56)	​	0.98 (0.79–1.00)	​
​	MMSP	1	0.47 (0.36–0.59)	​	0.98 (0.75–1.00)	​
​	PCR	1	0.64 (0.50–0.76)	​	0.99 (0.86–1.00)	​
​	RT-PCR	2	0.69 (0.38, 0.90)	​	0.92 (0.53, 0.99)	​
Multifocality	​	​	​	0.653	​	0.621
​	Solitary	4	0.43 (0.38–0.48)	​	0.91 (0.85, 0.95)	​
​	Multiple	26	0.46 (0.40, 0.52)	​	0.96 (0.93, 0.97)	​
​	Unknown	6	0.43 (0.37, 0.49)	​	0.94 (0.88, 0.97)	​

BALF, bronchoalveolar lavage fluid; BA, Bronchial aspirates; BW, bronchial washings; PE, pleural effusion; BEC, bronchial epithelial cells; FFPE, formalin-fixed paraffin-embedded; MSP, Methylation-Specific Polymerase Chain Reaction; qPCR, Quantitative Polymerase Chain Reaction; GLAD-PCR, Genomic Loci Allele Discrimination Polymerase Chain Reaction; QMSP, Quantitative Methylation-Specific Polymerase Chain Reaction; ddPCR, Droplet Digital PCR; MMSP, Multiplex methylation specific PCR; RT-PCR: PCR, Polymerase Chain Reaction; RT-PCR, Reverse Transcription Polymerase Chain Reaction.

Bold values indicate statistical significance (P < 0.05).

### 3.9 Leave-one-out sensitivity analysis

Leave-one-out sensitivity analyses were performed by sequentially excluding each study to evaluate the robustness of the pooled diagnostic estimates. For combined SHOX2/RASSF1A methylation, pooled sensitivity ranged from 0.766 to 0.787 and pooled specificity from 0.887 to 0.896 (Supplementary Table S2). For SHOX2 methylation, pooled sensitivity ranged from 0.678 to 0.706 and pooled specificity from 0.912 to 0.921 (Supplementary Table S3). For RASSF1A methylation, pooled sensitivity ranged from 0.447 to 0.463 and pooled specificity from 0.936 to 0.940 (Supplementary Table S4). Overall, only minimal changes in the pooled estimates were observed after sequential omission of individual studies, indicating that no single study had a substantial influence on the overall diagnostic performance and confirming the robustness of the findings.

## 4 Discussion

This systematic review and meta-analysis comprehensively evaluated the diagnostic performance of SHOX2 methylation, RASSF1A methylation, and their combined panel for lung cancer detection based on 49 eligible studies. The combined SHOX2/RASSF1A methylation panel demonstrated a pooled sensitivity of 0.778 (0.723–0.825) and specificity of 0.890 (0.866–0.911), with an HSROC AUC of 0.916, indicating excellent overall diagnostic performance. In comparison, SHOX2 methylation alone achieved higher sensitivity than RASSF1A methylation (0.694 vs. 0.457), whereas RASSF1A demonstrated the highest specificity (0.938). Pairwise comparisons further showed that the combined panel significantly improved sensitivity compared with either SHOX2 or RASSF1A alone while maintaining specificity comparable to SHOX2, although lower than that of RASSF1A. These findings suggest that combining SHOX2 and RASSF1A provides a more balanced diagnostic strategy than either individual biomarker and supports its potential role as an adjunctive biomarker for lung cancer detection.

DNA methylation is a stable epigenetic alteration that occurs early in tumorigenesis and can be detected in blood, sputum, and other body fluids (12). These properties make it an attractive biomarker for cancer detection. Our findings highlight the critical role of DNA methylation biomarkers, particularly *SHOX2* and *RASSF1A*, in lung cancer diagnosis. *SHOX2* and *RASSF1A* are well-established tumor suppressor genes implicated in the pathogenesis of various cancers, including lung cancer. *SHOX2*, located at chromosome 3q25.32, is a key regulator of organ development during embryogenesis and is frequently hypermethylated in lung cancer, resulting in gene silencing (22). Similarly, *RASSF1A* is a tumor suppressor gene commonly hypermethylated in lung, kidney, and other solid tumors, contributing to tumor initiation and progression (23). The combined methylation panel demonstrated improved diagnostic accuracy, highlighting the complementary diagnostic potential of *SHOX2* and *RASSF1A*. The clinical implications of our findings are significant. *SHOX2* and *RASSF1A* methylation has the potential to serve as a non-invasive diagnostic tool for lung cancer, particularly in patients where invasive biopsy procedures are not feasible or yield insufficient material. Moreover, its balanced sensitivity and specificity suggest that it can minimize false-positive diagnoses, reducing unnecessary follow-up procedures and anxiety for patients.

Our results align with earlier meta-analyses that reported the diagnostic value of *SHOX2* methylation in lung cancer detection. Zhao et al. demonstrated a sensitivity of 70% and specificity of 96% for *SHOX2* methylation, which is comparable to our findings. However, our study expands on these findings by incorporating *RASSF1A* methylation and exploring the combined diagnostic efficacy of both biomarkers. Interestingly, while *SHOX2* demonstrated higher sensitivity, *RASSF1A* displayed superior specificity. The combination of these biomarkers achieved an optimal balance between sensitivity and specificity, making it more suitable for clinical applications where both parameters are critical. Unlike previous meta-analyses that evaluated individual methylation markers separately, the present study directly compared pooled sensitivity and specificity among combined SHOX2/RASSF1A, SHOX2 alone, and RASSF1A alone using a joint bivariate meta-regression framework.

The combined analysis of SHOX2 and RASSF1A methylation represents a promising molecular diagnostic approach for lung cancer and may have potential utility in the early detection setting. However, because most included studies evaluated patients across mixed disease stages and did not report stage-specific diagnostic accuracy, the present meta-analysis could not assess performance specifically in early-stage disease. Its non-invasive nature nevertheless supports further evaluation in high-risk populations, such as smokers and individuals with a family history of lung cancer, particularly as an adjunct to existing screening strategies. Additionally, the ability to detect methylation in various sample types, including blood and sputum, enhances its feasibility for widespread clinical implementation. Future efforts should focus on standardizing methylation detection methods to ensure reproducibility and reliability across laboratories. Moreover, integrating methylation biomarkers with imaging modalities or other molecular markers could further improve diagnostic accuracy and guide personalized treatment strategies.

Although numerous DNA methylation biomarkers have been reported for lung cancer diagnosis, only SHOX2, RASSF1A, and their combined panel were supported by a sufficient number of independent studies with extractable diagnostic accuracy data to permit robust quantitative synthesis. Therefore, the present meta-analysis focused on these biomarkers. Despite prespecified subgroup analyses, substantial residual heterogeneity remained. One potential contributor to the residual heterogeneity is the variability in control populations across studies, which ranged from healthy individuals to patients with benign pulmonary diseases, atypical adenomatous hyperplasia, benign pleural effusion, and other non-malignant conditions. Studies using only healthy controls may overestimate diagnostic specificity compared with those including patients with benign pulmonary diseases or other malignancies, which more closely resemble real-world clinical diagnostic settings. Because many studies used mixed control populations or reported insufficient clinical details, subgroup analyses according to control type were not feasible. In addition, variations in methylation assays, laboratory protocols, and positivity definitions across studies may also have contributed to residual heterogeneity, although no significant threshold effect was detected. Together, these factors should be considered when interpreting the pooled diagnostic performance of SHOX2 and RASSF1A methylation. Future studies should adopt standardized methylation assays and prespecified positivity thresholds to improve comparability across studies and reduce between-study heterogeneity.

Despite these encouraging findings, several limitations should be acknowledged. First, most included studies were retrospective case-control studies, which may have overestimated diagnostic accuracy because of spectrum and selection bias. Second, substantial between-study heterogeneity remained despite subgroup analyses, likely reflecting differences in specimen type, assay methodology, and study populations. Third, significant publication bias was detected by Deeks’ funnel plot asymmetry test, suggesting that the pooled diagnostic performance may have been overestimated and should therefore be interpreted with appropriate caution. Finally, because most included studies enrolled patients across mixed disease stages and histological subtypes, stage-specific and histology-specific diagnostic performance could not be reliably evaluated. Consequently, the present findings reflect the overall diagnostic performance of these biomarkers across heterogeneous lung cancer populations rather than their accuracy in specific clinical settings. Future large-scale prospective studies using standardized methylation assays and uniform reporting of stage- and histology-specific diagnostic outcomes are warranted. In addition, cost-effectiveness analyses will be important before these biomarkers can be routinely implemented in clinical practice.

## 5 Conclusion

In summary, compared with either biomarker alone, the combined SHOX2/RASSF1A methylation panel significantly improved pooled sensitivity while maintaining high diagnostic specificity, supporting a more balanced overall diagnostic performance for lung cancer detection. These findings support its potential role as an adjunctive diagnostic approach, particularly for non-tissue or minimally invasive specimens. Nevertheless, large-scale prospective studies using standardized assay protocols are warranted before routine clinical implementation. Further studies should also evaluate stage-specific diagnostic performance, particularly in patients with stage I/II lung cancer, to better define the role of SHOX2/RASSF1A methylation in early detection.

## Data Availability

The datasets presented in this study can be found in online repositories. The names of the repository/repositories and accession number(s) can be found in the article/Supplementary Material.
