# Supplementary material for: Clinical value of combined SHOX2 and RASSF1A methylation in lung cancer diagnosis across tissue and liquid biopsy samples: a systematic review and meta-analysis
Source: Oncol Rev. 2026 Aug 28;20:1876563. doi: 10.3389/or.2026.1876563 (PMC13562240; doi:10.3389/or.2026.1876563)
Supplement: Supplementary file 1 [file Supplementaryfile1.docx]

**Supplementary Material 1. DNA methylation markers for lung cancer**

| **HUGO Acronyma^a^** | **Gene Name^b^** | **GO Annotation^c^** |
| --- | --- | --- |
| RASSF1A | Ras Association Domain Family Member 1 | Identical protein binding and obsolete protein N-terminus binding |
| SHOX2 | SHOX Homeobox 2 | Sequence-specific DNA binding |
| CDKN2A | Cyclin Dependent Kinase Inhibitor 2A | RNA binding and transcription factor binding |
| CDX2 | Caudal Type Homeobox 2 | DNA-binding transcription factor activity and transcription corepressor activity |
| HOXA1 | Homeobox A1 | Sequence-specific DNA binding |
| OPCML | Opioid Binding Protein/Cell Adhesion Molecule Like | G protein-coupled opioid receptor activity |
| NEUROG2 | Neurogenin 2 | Sequence-specific DNA binding and e-box binding |
| NID2 | Nidogen 2 | Calcium ion binding and collagen binding |
| APC | APC Regulator Of WNT Signaling Pathway | Binding and microtubule binding |
| HOXC9 | HOXC9 | Sequence-specific DNA binding |
| CDH1 | Cadherin 1 | calcium ion binding and protein phosphatase binding |
| MLH1 | MLH1 | ATP hydrolysis activity and mismatched DNA binding |
| RAR β | Retinoic Acid Receptor Beta | DNA-binding transcription factor activity and protein-containing complex binding |
| MGMT | O-6-Methylguanine-DNA Methyltransferase | Calcium ion binding and damaged DNA binding |
| DAPK | Death Associated Protein Kinase 1 | Identical protein binding and protein kinase activity |
| PAX5 | Paired Box 5 | DNA-binding transcription factor activity and DNA-binding transcription activator activity, rnapolymerase ii-specific |
| Dal-1 | Erythrocyte Membrane Protein Band 4.1 Like 3 | Actin binding and structural constituent of cytoskeleton |
| GATA5 | GATA Binding Protein 5 | DNA-binding transcription factor activity and transcription cis-regulatory region binding |
| SULF2 | Sulfatase 2 | Calcium ion binding and arylsulfatase activity |
| CXCL14 | C-X-C Motif Chemokine Ligand 14 | Chemokine activity |
| BRMS1 | BRMS1 Transcriptional Repressor And Anoikis Regulator | Histone deacetylase binding and histone deacetylase activity |
| PITX2 | Paired Like Homeodomain 2 | DNA-binding transcription factor activity and sequence-specific DNA binding |
| CDO1 | Cysteine Dioxygenase Type 1 | iron ion binding and cysteamine dioxygenase activity |
| HOXA9 | Homeobox A9 | Sequence-specific DNA binding and enzyme binding |
| AJAP1 | Adherens Junctions Associated Protein 1 |  |
| PTGDR | Prostaglandin D2 Receptor | G protein-coupled receptor activity and prostaglandin d receptor activity |
| UNCX | UNC Homeobox | Sequence-specific DNA binding |
| MARCH11 | Membrane Associated Ring-CH-Type Finger 11 |  |
| SOX17 | SRY-Box Transcription Factor 17 | DNA-binding transcription factor activity and protein heterodimerization activity |
| TAC1 | TAC1 | Substance p receptor binding |
| HOXA7 | Homeobox A7 | DNA-binding transcription factor activity and transcription factor binding |
| ZFP42 | ZFP42 Zinc Finger Protein | DNA-binding transcription factor activity and sequence-specific DNA binding |
| B3GAT2 | Beta-1,3-Glucuronyltransferase 2 | Galactosylgalactosylxylosylprotein 3-beta-glucuronosyltransferase activity |
| BCAR1 | BCAR1 Scaffold Protein, Cas Family Member | Protein kinase binding and sh3 domain binding |
| HLF | HLF Transcription Factor, PAR BZIP Family Member | DNA-binding transcription factor activity and double-stranded DNA binding |
| HOPX | HOP Homeobox |  |
| HOXD11 | Homeobox D11 | Sequence-specific DNA binding |
| MIR1203 | MicroRNA 1203 |  |
| MYL9 | Myosin Light Chain 9 | Calcium ion binding and structural constituent of muscle |
| SLC9A3R2 | NHERF Family PDZ Scaffold Protein 2 |  |

^a^Human Genome Organization nomenclature; ^b^Approved gene name from Human Genome Organization website <http://www.genenames.org/;> ^c^Gene Ontology (GO) annotations from GeneCards website.
