# Supplementary material for: Clinical value of combined SHOX2 and RASSF1A methylation in lung cancer diagnosis across tissue and liquid biopsy samples: a systematic review and meta-analysis
Source: Oncol Rev. 2026 Aug 28;20:1876563. doi: 10.3389/or.2026.1876563 (PMC13562240; doi:10.3389/or.2026.1876563)
Supplement: Supplementary file 2 [file Supplementaryfile3.docx]

**Supplementary Table 1. Heterogeneity statistics for the primary diagnostic accuracy meta-analysis**

| **Biomarker** | **Metric (95%CI)** | | **Q statistic** | **P (Q-test)** | **I² (%)** |
| --- | --- | --- | --- | --- | --- |
| SHOX2 &RASSF1A | Sensitivity | 0.778 (0.723-0.825) | 119.49 | <0.001 | 87.29 |
|  | Specificity | 0.890 (0.866-0.911) | 30.48 | 0.0625 | 26.98 |
|  | PLR | 7.102 (5.565-9.063) | 43.56 | 0.0017 | 47.2 |
|  | NLR | 0.249 (0.196-0.318) | 131.54 | <0.001 | 86.98 |
|  | DOR | 28.483 (18.095-44.835) | 81.54 | <0.001 | 73.44 |
| SHOX2 | Sensitivity | 0.694 (0.63-0.752) | 173.37 | <0.001 | 91.96 |
|  | Specificity | 0.917 (0.9-0.931) | 25.77 | 0.3117 | 19.14 |
|  | PLR | 8.363 (6.584-10.623) | 39.49 | 0.0175 | 44.1 |
|  | NLR | 0.334 (0.271-0.411) | 182.99 | <0.001 | 92.2 |
|  | DOR | 25.071 (16.468-38.166) | 69.82 | <0.001 | 67.03 |
| RASSF1A | Sensitivity | 0.457 (0.416-0.498) | 178.65 | <0.001 | 83.32 |
|  | Specificity | 0.938 (0.918-0.953) | 107.6 | <0.001 | 66.69 |
|  | PLR | 7.338 (5.624-9.573) | 100.25 | <0.001 | 57.42 |
|  | NLR | 0.579 (0.54-0.622) | 162.2 | <0.001 | 77.77 |
|  | DOR | 12.666 (9.474-16.934) | 78.19 | <0.001 | 50.36 |

**SupplementaryTable 2. Leave-one-out sensitivity analysis for the diagnostic accuracy of combined SHOX2 and RASSF1A methylation.**

| **Biomarker** | **Study omitted** | **Sensitivity (95%CI)** | **Specificity (95%CI)** |
| --- | --- | --- | --- |
| SHOX2&RASSF1A | Zhao (2024) | 0.785 (0.728-0.832) | 0.891 (0.865-0.912) |
|  | Ren (2017) | 0.782 (0.724-0.831) | 0.889 (0.862-0.911) |
|  | Zhong (2023) | 0.781 (0.723-0.830) | 0.889 (0.862-0.910) |
|  | Zhang (2023)A | 0.768 (0.715-0.814) | 0.887 (0.862-0.907) |
|  | Zhang (2023)B | 0.782 (0.723-0.831) | 0.892 (0.867-0.914) |
|  | Zhang (2017) | 0.777 (0.718-0.827) | 0.888 (0.863-0.909) |
|  | Shi (2020) | 0.768 (0.713-0.815) | 0.894 (0.867-0.916) |
|  | Lu (2023) | 0.774 (0.717-0.823) | 0.894 (0.868-0.915) |
|  | Liu (2024) | 0.770 (0.714-0.819) | 0.890 (0.865-0.912) |
|  | Liang (2022) | 0.781 (0.723-0.830) | 0.889 (0.863-0.910) |
|  | Jin (2024 a) | 0.783 (0.726-0.831) | 0.891 (0.866-0.912) |
|  | Jin (2024 b) | 0.785 (0.729-0.831) | 0.891 (0.866-0.912) |
|  | Gao (2022 a) | 0.781 (0.723-0.830) | 0.890 (0.865-0.911) |
|  | Gao (2022 b) | 0.779 (0.721-0.828) | 0.889 (0.864-0.910) |
|  | Chen (2023) | 0.782 (0.725-0.830) | 0.893 (0.868-0.913) |
|  | Xiang (2023) | 0.787 (0.738-0.830) | 0.896 (0.873-0.914) |
|  | Xie (2024) | 0.777 (0.718-0.826) | 0.893 (0.868-0.914) |
|  | Chen (2025) | 0.779 (0.721-0.828) | 0.893 (0.868-0.914) |
|  | Zeng(2025) | 0.783 (0.726-0.831) | 0.891 (0.866-0.912) |
|  | Gu (2025 a) | 0.768 (0.714-0.815) | 0.887 (0.862-0.908) |
|  | Gu (2025 b) | 0.766 (0.714-0.811) | 0.887 (0.863-0.908) |

**Supplementary Table 3. Leave-one-out sensitivity analysis for the diagnostic accuracy of SHOX2 methylation.**

| **Biomarker** | **Study omitted** | **Sensitivity (95%CI)** | **Specificity (95%CI)** |
| --- | --- | --- | --- |
| SHOX2 | Xu (2020) | 0.696 (0.629-0.757) | 0.919 (0.901-0.934) |
|  | Vo (2022) | 0.699 (0.632-0.758) | 0.918 (0.900-0.933) |
|  | Schmidt (2010) | 0.696 (0.628-0.757) | 0.912 (0.894-0.927) |
|  | Kneip (2011 a) | 0.699 (0.632-0.758) | 0.921 (0.904-0.935) |
|  | Kneip (2011 b) | 0.693 (0.626-0.753) | 0.917 (0.900-0.931) |
|  | Huang (2020 a) | 0.698 (0.631-0.758) | 0.917 (0.899-0.931) |
|  | Huang (2020 b) | 0.697 (0.630-0.756) | 0.918 (0.901-0.932) |
|  | Feng (2020) | 0.678 (0.618-0.733) | 0.915 (0.898-0.930) |
|  | Dietrich (2012) | 0.690 (0.623-0.751) | 0.914 (0.897-0.928) |
|  | Zhao (2024) | 0.702 (0.637-0.760) | 0.917 (0.900-0.932) |
|  | Ren (2017) | 0.697 (0.629-0.757) | 0.917 (0.899-0.932) |
|  | Zhang (2023) A | 0.683 (0.620-0.740) | 0.915 (0.898-0.929) |
|  | Zhang (2023) B | 0.697 (0.629-0.757) | 0.919 (0.901-0.933) |
|  | Shi (2020) | 0.690 (0.622-0.750) | 0.918 (0.900-0.933) |
|  | Lu (2023) | 0.692 (0.625-0.752) | 0.919 (0.901-0.933) |
|  | Liu (2024) | 0.690 (0.622-0.750) | 0.916 (0.899-0.931) |
|  | Liang (2022) | 0.700 (0.634-0.759) | 0.917 (0.900-0.931) |
|  | Jin (2024) | 0.699 (0.633-0.758) | 0.917 (0.900-0.932) |
|  | Gao (2022) | 0.701 (0.636-0.759) | 0.917 (0.900-0.932) |
|  | Xiang (2023) | 0.706 (0.648-0.758) | 0.919 (0.901-0.934) |
|  | Xie (2024) | 0.694 (0.627-0.755) | 0.920 (0.902-0.934) |
|  | Chen (2025) | 0.693 (0.626-0.753) | 0.919 (0.901-0.933) |
|  | Gu (2025 a) | 0.685 (0.620-0.744) | 0.916 (0.898-0.930) |
|  | Gu (2025 b) | 0.681 (0.619-0.738) | 0.915 (0.898-0.929) |

**Supplementary Table 4. Leave-one-out sensitivity analysis for the diagnostic accuracy of RASSF1A methylation.**

| **Biomarker** | **Study omitted** | **Sensitivity (95%CI)** | **Specificity (95%CI)** |
| --- | --- | --- | --- |
| RASSF1A | Yu (2008) | 0.461 (0.420-0.502) | 0.936 (0.916-0.952) |
|  | Shivapurkar (2007) | 0.457 (0.415-0.499) | 0.937 (0.916-0.952) |
|  | Smetannikova (2019) | 0.455 (0.414-0.497) | 0.938 (0.917-0.954) |
|  | Shah (2020) | 0.458 (0.416-0.500) | 0.937 (0.916-0.953) |
|  | Wang (2007) | 0.460 (0.419-0.502) | 0.936 (0.916-0.952) |
|  | Schmiemann (2005) | 0.458 (0.416-0.500) | 0.936 (0.915-0.952) |
|  | Rykova (2004) | 0.457 (0.416-0.499) | 0.937 (0.917-0.953) |
|  | Roncarati (2020) | 0.457 (0.415-0.499) | 0.937 (0.917-0.953) |
|  | Ponomaryova (2013) | 0.451 (0.411-0.492) | 0.939 (0.924-0.952) |
|  | Peng (2010) | 0.454 (0.413-0.496) | 0.937 (0.917-0.953) |
|  | Lewandoska (2015) | 0.462 (0.422-0.503) | 0.938 (0.918-0.954) |
|  | Nunes (2019) | 0.463 (0.424-0.503) | 0.938 (0.917-0.953) |
|  | Nawaz (2014) | 0.456 (0.415-0.499) | 0.937 (0.917-0.953) |
|  | Mohammed (2022) | 0.456 (0.415-0.499) | 0.940 (0.921-0.955) |
|  | Ma (2016) | 0.453 (0.412-0.494) | 0.937 (0.916-0.953) |
|  | Liu (2007) | 0.457 (0.416-0.500) | 0.937 (0.916-0.953) |
|  | Kim (2004) | 0.459 (0.417-0.501) | 0.937 (0.917-0.953) |
|  | Hubers (2014 a) | 0.458 (0.416-0.500) | 0.938 (0.917-0.954) |
|  | Hubers (2014 b) | 0.455 (0.414-0.497) | 0.938 (0.917-0.954) |
|  | Hubers (2016) | 0.461 (0.421-0.502) | 0.939 (0.918-0.955) |
|  | Hubers (2015 a) | 0.458 (0.416-0.500) | 0.937 (0.916-0.953) |
|  | Hubers (2015 b) | 0.460 (0.419-0.502) | 0.940 (0.920-0.955) |
|  | Grote (2006) | 0.456 (0.414-0.498) | 0.937 (0.916-0.952) |
|  | Gao (2015) | 0.452 (0.412-0.493) | 0.940 (0.921-0.955) |
|  | Constâncio (2019) | 0.463 (0.423-0.503) | 0.938 (0.917-0.954) |
|  | Hubers (2012) | 0.461 (0.420-0.502) | 0.937 (0.916-0.953) |
|  | Zhao (2024) | 0.457 (0.415-0.499) | 0.939 (0.918-0.955) |
|  | Ren (2017) | 0.455 (0.414-0.498) | 0.937 (0.916-0.953) |
|  | Zhang (2023) A | 0.453 (0.412-0.494) | 0.937 (0.917-0.953) |
|  | Zhang (2023) B | 0.457 (0.416-0.500) | 0.938 (0.918-0.954) |
|  | Shi (2020) | 0.452 (0.412-0.493) | 0.936 (0.915-0.952) |
|  | Lu (2023) | 0.457 (0.416-0.499) | 0.938 (0.918-0.954) |
|  | Liu (2024) | 0.457 (0.415-0.499) | 0.937 (0.917-0.953) |
|  | Liang (2022) | 0.457 (0.416-0.499) | 0.937 (0.916-0.953) |
|  | Jin (2024) | 0.452 (0.412-0.493) | 0.939 (0.919-0.955) |
|  | Gao (2022) | 0.458 (0.417-0.500) | 0.937 (0.916-0.952) |
|  | Xiang (2023) | 0.458 (0.416-0.500) | 0.940 (0.920-0.955) |
|  | Xie (2024) | 0.447 (0.411-0.484) | 0.940 (0.921-0.955) |
|  | Chen (2025) | 0.459 (0.418-0.501) | 0.938 (0.917-0.954) |
|  | Gu (2025 a) | 0.455 (0.413-0.497) | 0.937 (0.917-0.953) |
|  | Gu (2025 b) | 0.455 (0.413-0.496) | 0.938 (0.917-0.953) |
